# Comparative Analysis of Transverse and Longitudinal Ultrasound Techniques for Cricothyroid Membrane Identification in Anesthesiology Trainees

**DOI:** 10.7759/cureus.83690

**Published:** 2025-05-07

**Authors:** Melissa Lourdes Carlos, Pushpraj Singh, Asif Dabeer Jafri, Utsav Anand Mani, Shyam Sundar

**Affiliations:** 1 Anesthesiology, P.D. Hinduja Hospital and Medical Research Centre, Mumbai, IND; 2 Anesthesiology, Career Institute of Medical Sciences, Lucknow, IND; 3 Emergency Medicine, Sanjay Gandhi Post Graduate Institute of Medical Sciences, Lucknow, IND; 4 Internal Medicine, Sum Ultimate Medicare, Bhubaneswar, IND

**Keywords:** anesthesiology trainees, cricothyroid membrane, cricothyrotomy, emergency airway management, longitudinal approach, transverse approach, ultrasound-guided identification

## Abstract

Background and objectives

Cricothyrotomy is a life-saving procedure in "cannot intubate, cannot ventilate" scenarios, with accurate identification of the cricothyroid membrane (CTM) being critical to success. Palpation techniques are often unreliable, especially in obese patients. This study aimed to compare the time taken and success rates of transverse versus longitudinal ultrasound approaches for CTM identification by anesthesiology trainees in volunteers of varying body habitus.

Methods

In this prospective, randomized, crossover study, 55 novice anesthesiology residents received a brief training session, followed by ultrasound-guided CTM identification on slender, overweight, and obese male volunteers using both transverse and longitudinal techniques. Each attempt was timed and confirmed by an expert anesthesiologist. Statistical analyses included the Wilcoxon signed-rank test for time comparisons and the Cochran Q test for success rates, with a success threshold defined as CTM identification within 120 seconds.

Results

The transverse approach demonstrated similar identification times to the longitudinal approach in slender (64.5±46.5 sec vs. 67.7±46.8 sec) and overweight (76.4±54.7 sec vs. 67.7±46.8 sec) volunteers (p>0.05), but was significantly faster in obese volunteers (88.4±59 sec vs. 93.8±64.8 sec, p=0.011). Success rates within 120 seconds were high across all groups: 100% in slender, 98.2% in overweight, and 96.3% in obese volunteers, with no significant difference between techniques, though the transverse approach showed a slight advantage in obese subjects.

Conclusions

Both ultrasound techniques are effective for CTM identification by novice anesthesiology residents. The transverse approach offers a time advantage in obese patients. High success rates were achieved following brief training, supporting the integration of ultrasound into emergency airway management protocols, particularly for anticipated difficult airways.

## Introduction

Cricothyrotomy is a critical, last-resort airway management technique employed in emergency situations when conventional methods of airway access, such as orotracheal or nasotracheal intubation, are unsuccessful. It is typically indicated in scenarios of "cannot intubate, cannot ventilate," when oxygen saturation (SpO₂) cannot be maintained above 90%, or in cases involving maxillofacial trauma that precludes oral or nasal access. The cricothyroid membrane (CTM), situated between the thyroid and cricoid cartilages in the anterior midline of the neck, is the target site for this procedure. Anatomically, it is approximately 2-3 cm below the thyroid cartilage prominence and varies in size, ranging from 8 to 19 mm in the superoinferior dimension and 9 to 19 mm laterally, as determined in adult cadaveric studies [1].

The CTM is an ideal access point for emergency airway interventions due to its superficial location, relative avascularity, and the absence of significant structures directly posterior to it. Traditional identification techniques include visual inspection and palpation; however, these methods become unreliable in obese individuals, where anatomical landmarks are obscured by adipose tissue. Consequently, there has been an increasing emphasis on the use of ultrasonography for CTM identification, especially in patients with anticipated difficult airways.

Recent airway management guidelines recommend the use of pre-procedural ultrasound to delineate neck anatomy and mark the CTM, aiming to improve cricothyrotomy success and reduce complications. Two ultrasound techniques, the transverse and longitudinal approaches, have been described and are reproducible with a high degree of accuracy in CTM identification compared to palpation [2-3]. For instance, the longitudinal technique has demonstrated a median time of 48 seconds to identify the CTM in morbidly obese patients, reinforcing its utility in pre-intubation planning [4].

There remains a clinical need for a rapid, reliable method for CTM identification that is applicable in emergency settings and can be adopted by ultrasound-naïve anesthesiologists after brief training [5]. The primary objective of this study was to compare the time taken by anesthesiology trainees to identify the CTM using transverse and longitudinal ultrasound approaches in overweight and obese male volunteers. The secondary objective was to compare the success rates of these techniques following minimal training.

## Materials and methods

This prospective, randomized, crossover study was conducted over 12 months at a tertiary care academic hospital. The study population consisted of junior anesthesiology residents enrolled in the Department of Anesthesiology. Institutional Ethics Committee of Seth G. S. Medical College and KEM Hospital, Mumbai issued approval IEC-Il reference No. EC/32/2018 before commencement, and written informed consent was obtained from all participants.

Inclusion and exclusion criteria

Eligible participants included junior residents in their 1st, 2nd, or 3rd year of MD or Diploma training in Anesthesiology who had no prior experience using ultrasound to identify the CTM. Residents with physical limitations precluding the use of ultrasound or prior practical experience in airway ultrasonography were excluded.

Three male volunteers were selected for ultrasound scanning, representing different body habitus categories based on BMI: slender (BMI < 24.9), overweight (BMI 25-29.9), and obese (BMI > 30).

Study design and procedure

Participants were randomized into two groups using computer-generated random numbers to determine the order of technique application - either starting with the transverse or longitudinal ultrasound approach. All participants viewed a six-minute instructional video demonstrating neck anatomy, ultrasound probe positioning, and technique for CTM identification. This was followed by a 10-minute supervised hands-on practice session on a slender male volunteer.

In the transverse approach, the transducer was placed transversely on the anterior neck to identify the thyroid cartilage, cricoid cartilage, and CTM, which was then marked with a skin marker. In the longitudinal approach, the transducer was placed in the midline longitudinally to visualize the tracheal rings and cricoid cartilage, with the CTM identified and similarly marked.

After a one-hour interval post-training, participants attempted CTM identification on the overweight and obese male volunteers using both techniques in a crossover manner. Each attempt was limited to 240 seconds. An expert anesthesiologist verified the accuracy of each identification immediately after the attempt.

A standardized ultrasound machine (specific model and settings consistent throughout the study) was used by all participants, and the same volunteers were used for all procedures to minimize variability.

Sample size and statistical analysis

Sample size calculation was based on previous studies, aiming to detect a 10% difference in time between the two techniques. A total of 55 participants were required to achieve a statistical power of 80% with a significance level of 5%. Data analysis included the Wilcoxon signed-rank test for paired comparison of time between techniques and the Cochran Q test for evaluating differences in success rates across volunteer categories. A p-value < 0.05 was considered statistically significant.

## Results

A total of 55 anesthesiology residents participated in the study, with a mean age of 27.07 ± 1.75 years (range: 25-34 years). The cohort comprised 67.3% females (n=37) and 32.7% males (n=18). The majority of participants (81.8%, n=45) were pursuing a Doctor of Medicine (MD) degree, while 18.2% (n=10) were enrolled in a Diploma in Anesthesiology (DA) program. Distribution by year of residency showed 29.1% (n=16) were first-year residents, 45.5% (n=25) second-year, and 25.5% (n=14) third-year. All participants completed the standardized training video and hands-on session prior to the assessment. Regarding the sequence of techniques used, 50.9% (n=28) initially performed the longitudinal approach, and 49.1% (n=27) started with the transverse approach. Table 1 presents the comparison of time taken to identify the cricothyroid membrane (CTM) using transverse and longitudinal approaches across three volunteer categories: slender, overweight, and obese.

**Table 1:** Time taken (in seconds) to identify the cricothyroid membrane using transverse and longitudinal ultrasound approaches. The data presented includes mean ± standard deviation (SD) for each approach, along with the p-value derived from the Wilcoxon signed-rank test. Z-values represent the test statistics.

Volunteer Type	Transverse Approach (Mean ± SD)	Longitudinal Approach (Mean ± SD)	P-value (Wilcoxon signed-rank test)	Test Statistic	Interpretation
Slender	64.5 ± 46.5	67.7 ± 46.8	0.068	Z= -1.822	No significant difference
Overweight	76.4 ± 54.7	79.6 ± 59.0	0.067	Z= -1.832	No significant difference
Obese	88.4 ± 59.0	93.8 ± 64.6	0.011	Z = -2.53	Transverse approach significantly faster

Further analysis using Friedman’s test revealed a statistically significant difference in identification times across the three BMI categories for both techniques (p<0.001). Post hoc pairwise comparisons using Wilcoxon signed-rank test with Bonferroni correction demonstrated that identification time increased significantly from slender to overweight (p<0.001), slender to obese (p<0.001), and overweight to obese volunteer (p<0.001) for both approaches. Success rates for CTM identification at 120 seconds and 240 seconds using both approaches are presented in Table 2.

**Table 2:** Success rates for CTM identification using transverse and longitudinal ultrasound approaches. Statistical tests used for analyzing success rates include the Cochran Q test for both TA and LA. The success rates are presented as n (%) for each volunteer type at the given time intervals. TA: Transverse Approach; LA: Longitudinal Approach

Type of volunteer	TA: Success at 120 sec	TA: Success at 240 sec	TA: No Success at 240 sec	LA: Success at 120 sec	LA: Success at 240 sec	LA: No Success at 240 sec	Cochran Q (TA)	Cochran Q (LA)
Slender	49 (89.1%)	6 (10.9%)	-	49 (89.1%)	6 (10.9%)	-	0.015	0.004
Overweight	44 (80%)	10 (18.2%)	1 (1.8%)	44 (80%)	10 (18.2%)	1 (1.8%)	-	-
Obese	45 (81.8%)	8 (14.5%)	2 (3.6%)	42 (76.4%)	11 (20%)	2 (3.6%)	-	-

For the transverse approach, success at 120 seconds was highest in the slender volunteer (89.1%), followed by the obese (81.8%) and overweight volunteers (80%). Cochran’s Q test revealed a significant difference in success rates across the three volunteer categories (p=0.015). Post hoc analysis using McNemar’s test with Bonferroni correction showed no significant differences between slender and overweight volunteers (p=0.063), slender and obese volunteers (p=0.125), or overweight and obese volunteers (p=1.000).

For the longitudinal approach, success at 120 seconds was also highest in the slender volunteer (89.1%), followed by overweight (80%) and obese volunteers (76.4%). Cochran’s Q test again indicated a significant difference across the groups (p=0.004). Post hoc analysis demonstrated a statistically significant difference between slender and obese volunteers (p=0.016), while comparisons between slender and overweight (p=0.063) and between overweight and obese volunteers (p=0.500) were not significant. Hence, both ultrasound techniques showed significantly increased time to identify the CTM in higher BMI volunteers. The transverse approach was significantly faster than the longitudinal approach in the obese volunteer (p=0.011), with no significant time difference in slender or overweight volunteers. Success rates were consistently high across all groups but were lowest in the obese volunteer using the longitudinal approach. The longitudinal approach had a significantly higher success rate with the slender volunteer than with the obese volunteer.

## Discussion

For a successful cricothyroidotomy, accurate identification of the cricothyroid membrane (CTM) is critical. Several previous studies have documented that the accuracy of CTM identification by traditional palpation methods in obese patients is less than 40% [6-7]. The use of ultrasound has been increasing rapidly, and its accuracy in identifying the CTM is superior to traditional methods. This study was conducted at our tertiary care center to evaluate the time required by novice residents to identify the CTM using ultrasound. The aim was to compare the time and success rates of CTM identification using two ultrasound techniques: the longitudinal and transverse approaches. In the majority of previous studies, only one or two practitioners performed the ultrasound scans either as a descriptive or reference method [6-12]. With each scan, these practitioners potentially gained experience, introducing bias in favor of the ultrasound technique. In contrast, our study involved 55 novice anesthesiology residents who performed ultrasound scans to identify the CTM in slender, overweight, and obese male volunteers. Participants underwent a 6-minute instructional video followed by a 10-minute hands-on session with a slender male volunteer. After a one-hour waiting period, each resident was asked to mark the CTM using a skin marker on one overweight and one obese male volunteer, applying both ultrasound techniques. In a comparable study, Kristensen et al. trained 42 anesthesiologists with an hour-long structured program (including e-learning, a lecture, and hands-on training) and evaluated both techniques in a randomized, crossover design in obese female subjects [5].

The mean age of participants in our study was 27.07 ± 1.75 years (range 25-34), with 37 (67.3%) female and 18 (32.7%) male residents. Of the total, 10 (18.2%) were diploma residents and 45 (81.8%) were MD residents. The cohort included 16 first-year, 25 second-year, and 14 third-year residents. In comparison, Kristensen et al. studied 32 certified anesthesiologists and 10 residents in training [5]. Given the crossover design of our study, 27 residents first performed the transverse approach, followed by the longitudinal approach, while 28 performed them in the reverse order. The mean time to identify the CTM using the transverse approach in the slender volunteer was 64.5 ± 46.5 seconds, comparable to the longitudinal approach (67.7 ± 46.8 seconds). Similar results were observed in the overweight volunteer (p = 0.067). However, in the obese volunteer, the transverse approach (88.4 ± 59 seconds) was significantly faster than the longitudinal approach (93.8 ± 64.8 seconds; p = 0.011). Kristensen et al. reported similar findings, with the transverse technique being faster (mean 24.0 ± 12.4 seconds vs. 37.6 ± 17.9 seconds; p = 0.0003) [5].

Unlike other studies, we compared both the time taken and success rates for identifying the CTM in slender, overweight, and obese individuals. The time taken using the transverse approach significantly increased from slender (64.5 ± 46.5 seconds) to overweight (76.4 ± 54.7 seconds) and obese volunteers (88.4 ± 59 seconds). A similar trend was observed with the longitudinal approach, which showed increasing times in overweight (67.7 ± 46.8 seconds) and obese volunteers (93.8 ± 64.6 seconds) compared to the slender volunteer. In Kristensen et al.’s study, a 120-second threshold was used to define a failed attempt. Since our participants were trainees, we extended the failure threshold to 240 seconds. At the 120 second mark, success rates using the transverse approach were 49 (89.1%) in slender, 44 (80%) in overweight, and 45 (81.8%) in obese volunteers, while those for the longitudinal approach were 49 (89.1%) in slender, 44 (80%) in overweight, and 42 (76.4%) in obese volunteers. A significant difference in success rates was noted between slender and obese volunteers for the longitudinal approach (p = 0.016). Kristensen et al. reported a 90% success rate for both techniques [5]. Our study showed overall success rates of 55 (100%) in slender, 54 (98.2%) in overweight, and 53 (96.3%) in obese volunteers. These findings demonstrate that even with minimal training, novice residents can achieve clinically useful skills in CTM identification using ultrasound. Boet et al. found that cricothyrotomy skills can be retained for at least 12 months post-training [13].

Given the growing role of ultrasound in regional anesthesia, we recommend preoperative identification and marking of the CTM, especially in patients with anticipated difficult airways. Mallin et al. demonstrated that ultrasound-guided CTM markings remain accurate even after head manipulation during simulated intubation, supporting the value of pre-procedure identification [14]. With advances in technology and smaller probes, these findings may evolve. Our volunteers had no neck pathology, limiting the generalizability of our findings. Nonetheless, we suggest using the transverse approach in cases with short necks or limited transducer placement options, such as in patients with ankylosing spondylitis or radiation fibrosis. The longitudinal technique allows visualization of the midline trachea, cricotracheal space, and inter-tracheal spaces, which is useful in scenarios requiring emergency tracheal access rather than cricothyroidotomy, for example, in small children, tumors overlying the CTM, subglottic stenosis, elective tracheostomy, or retrograde intubation [15-17].

## Conclusions

The time required for identifying the cricothyroid membrane using ultrasound was comparable between the transverse and longitudinal approaches in slender and overweight male volunteers. However, in the obese volunteer, the transverse approach was significantly faster than the longitudinal approach. Both ultrasound techniques demonstrated a 100% success rate in identifying the cricothyroid membrane in the slender volunteer, with success rates of 98.2% in overweight and 96.3% in obese volunteers. These findings highlight the effectiveness of both techniques, with the transverse approach offering a faster identification, particularly in obese individuals.
